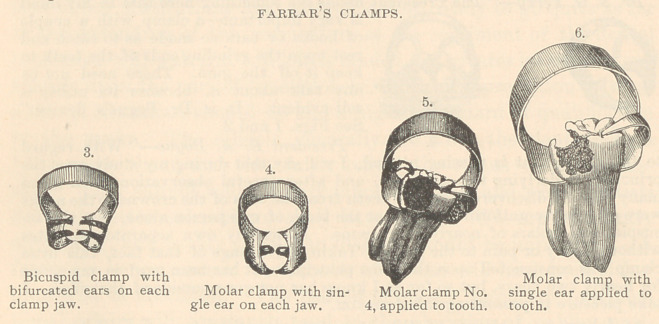# Current News and Opinion

**Published:** 1887-12

**Authors:** 


### Current News and Opinion.

### PAINLESS CLAMPS.

Among the proceedings of the New York Odontological Society of June 14, 1887, as published in the *Dental Cosmos* in October, I notice the following:

*Dr. S. G. Perry*—"The President hands me something here that to my mind is very important—a clamp with a couple of hooks or ears so made as to catch and rest upon the grinding ends of the teeth to keep it off the gum. There need not be any talk about it, because its utility is self-evident. It is Dr. Bogue's device." See Figs. 1 and 2.

1.

2.

*President E. A. Bogue*—"With regard to the clamp that is passing around, I will say that during my study over the principles underlying the separator, and after careful observations in a great many cases, I discovered that the teeth from the tops of the crowns to the necks were of a fairly uniform length; not the teeth of one person alone, but the bicuspids and molars of nearly all persons. Hence my own separator operates without injury or pain to the gums. Taking advantage of that fact, this little clamp was constructed upon the same principle. It has been used in my office for nearly two years, but so far as I know has not gone outside of the office. I take pleasure in presenting it before you."

As I have used clamps of this form for more than ten years (eight years longer than Dr. Bogue says he has), I wish to endorse Dr. Perry's remarks concerning their merits. These clamps are so valuable to me that I would not on any account dispense with them in my practice.

Probably there is no instrument that patients dread more than the ordinary clamp, which bites down and impinges upon the margin of the gums. The shape of many teeth renders it necessary for the jaws of the clamp to take a deep hold, and in some cases to extend even beneath the gum, in order that it may not slip off. Indeed, some teeth are so conical that no clamp will remain secure long enough to allow the completion of an operation. But there are other teeth with crowns so large and necks so small that the jaws of the ordinary clamp will imperceptibly creep down from the crown to the neck, and will encroach upon the gum sufficiently, in many cases, to cause excruciating pain, and even injury to the gum.

It is only necessary to use a clamp properly constructed, which, while perfectly efficient, cannot cause pain or injury by creeping. For the comfort of patients, and I may say of myself also, I have, as before said, used with great satisfaction a set of such clamps, and thinking that they might be useful to others, I published an account of them in the *Dental Cosmos*, April, 1877, pp. 288, 289. As the description there given is brief and clear, I will quote direct from the article:

"The avoidance of pain is secured by having an annealed ear, or two ears, projecting from the jaws of the clamp, so as to rest upon the grinding surface of the crown, and allow the jaws to remain at a desired fixed point on the side of the tooth without liability to creep down upon the margin of the gum.

"These painless clamps are made of different sizes and shapes to suit different teeth, some having one ear and others two; some suited to long crowns and others to short ones. From the set a clamp may be selected for each case, so shaped that the ear or ears will not be in the way The object in having the ears annealed is, that they may be bent to suit different lengths of crowns in rare emergencies; but if the operator has several sizes, there is little or no necessity for doing this. Although I use a set of a dozen of these clamps, more than six are not often necessary. If one is to be used on trial, the one-ear molar clamp is the most useful.

FARRAR'S CLAMPS.

3.

Bicuspid clamp with bifurcated ears on each clamp jaw.

4.

Molar clamp with single ear on each jaw.

5.

Molar clamp No. 4, applied to tooth.

6.

Molar clamp with single ear applied to tooth.

"The set consists of a right and a left one-ear (*i e.*, on one side only) molar clamp; a right and a left bicuspid clamp, with a bifurcated ear on one jaw; and two molar clamps, having an ear on each jaw, one of the clamps suited to long crowns, and the other to short crowns."

Figs. 3, 4, 5, 6 illustrate a sample of each variety of this set of clamps, which are of my own devising. The bicuspid clamp, with bifurcated ears on one clamp jaw only, does not appear in the illustration.

J. N. Farrar, M. D., D. D. S.

1271 Broadway, New York City.

---

### DENTAL SQUIBBS.

BY "MEANDER."

Hear what Professor Gross says: "Dentistry is the most important specialty in medicine. Many people come into the world and go out of it who never require the other specialties, but *no* child is born who does not, sooner or later, require the services of a dentist."

Now, suppose that child has the misfortune to come under the care of a mediocre dentist, whose greatest success lies in destroying live pulps and extracting teeth that can be easily saved to do good service for years. A good natural tooth is worth a whole mouthful of artificial ones, and if any man saves a natural tooth from destruction, he, indeed, does a great service and "will hide a multitude of sins."

There are cases in which it is excusable to destroy a pulp. But happily, they are few. It may be admissible in the almost total destruction of one or more of the crowns of the cuspids, or the lateral and central incisors. He who, in such cases, has removed the pulp from the root-canal by driving up a fine wooden stick, and then removed the tissue and immediately stopped the apical foramen with any suitable material, has found that abscess does *not* occur. Why?

He who has read Dr. Black's erudite and painstaking paper in this Journal, page 462, Vol. VIII, has discovered a good reason. It may be the true one. Hear what he says. Read it over several times! "If we have found the conditions favorable," note 'favorable,' "abscess following the removal of the tooth-pulp should be impossible. We may pass our broach through the apical foramen and wound and lacerate the tissues, and possibly provoke an inflammatory movement, but"—now notice what he says—"if the root-canal and the instrument be aseptic, abscess cannot occur! This I have tried, experimentally, in a sufficient number of instances to be convinced that it is practically true, as well as theoretically true. In several cases I have aroused a considerable degree of inflammation, but in no case did abscess occur." Why? "These experiments were made by broaches cleaned," that is, made aseptic "by heat."

Dr. Black's position is, that there must be no microbes either in the pulp-canal or on the broach. Both must be aseptic; that is, free from microbes.

How would it work if each operator should use extraordinary care to have his forceps, excavators, engine-burs, corundum disks, and all manner and forms of dental implements most thoroughly cleaned after using them upon a patient, and before they are applied to another patient? How many do this thing thoroughly? How many leave it to an indifferent assistant?

Does not this paper of Dr. Black set some of us thinking? And thinking, can we not benefit our patients by it? Can we not manage to stumble a little less? Suppose we had no dental literature, how many of us would have known this?

---

### THE TOOTH CROWN AND BRIDGE PATENTS.

NOVEMBER 1ST, 1887.

*A. L. Northrop, D. D. S.*

DEAR SIR:—In answer to your request on behalf of the first District Dental Society of New York, asking for our opinion as to the legal position of the dental profession, with regard to the crown and bridge patents of the "International Tooth Crown Company," in view of the recent decision of Judges Wallace and Shipman, in the Richmond and Gaylord suits, and advice as to relief from further claims made under the Low bridge patent, we have to say:

These suits involved the validity of the two patents to Cassius M. Richmond, Nos. 277,941 and 277,943, for "Tooth Crowns," etc., the patent to Alvan S. Richmond, No. 277,953, for "bridge," all dated May 22d, 1883, and the patent to James E. Low, for "method of supporting artificial teeth by bands cemented to permanent teeth," No. 238,940, dated March 15th, 1881.

The first two patents covered what is known as the "Richmond" and the "Sheffield" tooth crowns in all its varieties. They were held invalid, and therefore you are at liberty to make such tooth crowns without being in any way liable to the International Tooth Crown Co.

The complainants have appealed this case to the U. S. Supreme Court, but we do not advise you that any different decision will probably result. The practical result is that the tooth crown is free.

The patent for the Richmond bridge was also held invalid, but the Low patent was declared to be good. This Low patent covers a bridge attached to continuous bands cemented to adjoining permanent teeth, "whereby said artificial teeth are supported by said permanent teeth without dependence on the gum beneath."

The Richmond patent is, as you will remember, for a bridge supported by caps, and the Court held that it was not invention for Richmond to support a bridge on caps, but it was invention for Low to support a bridge on bands, taking all the surrounding circumstances into consideration, and that as a cap was nothing but a band with a roof on it, the Richmond bridge infringed the Low patent.

The practical effect of this decision, if the complainant chooses to follow it up diligently, and unless some new evidence is found, will be to shut the profession out from inserting permanent bridges supported at one or more points by cemented caps or bands without dependence on the gum.

As the matter now stands, any dentist inserting a Richmond bridge (according to the decision), infringes the Low patent; and an injunction would doubtless now be granted by any Federal Judge on application, on the strength of that adjudication alone.

An appeal can be taken by the defendants to the Supreme Court, a year or so hence, after an accounting by them, and determining the amount of profits or damages the complainant is entitled to recover.

The way of relief is for all the dentists of the United States, who supported artificial teeth on a band or bar, surrounding and extending between permanent teeth prior to September, 1878, to send to us at No. 833 Broadway, New York City, or to No. 9 Law Chambers, New Haven, Connecticut, a truthful description of what he did, and for whom, and where and when.

If such proofs can be made strong and clear enough to satisfy the Court that what Low described was well known, and had been long practiced by dentists in the United States before Low claims to have done it, the present case might be opened for re-hearing on the newly discovered evidence—or the Courts might refuse to grant injunctions, upon the ground that the present decision would have been the other way if this evidence had been before it — at any rate, the question of the validity of the Low patent would be re-tried, if its owner ever had the temerity to sue a dentist whose mouth had not been closed by a license, in which he covenanted never to deny its validity.

Whether, in a suit against such a licensee, the Court would enjoin upon the *covenants*, under a patent declared void, either before or after the taking of the license, we cannot say.

Your obediant servants,

Solomon J. Gordon,
833 Broadway, New York City.

John K. Beech,
9 Law Chambers, New Haven, Conn.

### THE CONGRESS FROM AN ENGLISH POINT OF VIEW.

All who were guests at Washington feel very cordially their indebtedness to their American fellow-professionals, and will for a very long while remember their hospitality and courteous bearing towards the "strangers." Among the sections our own specialty was very conspicuous; with 500 entries it was able to present better audiences and wider discussion than most of the other sections. If the communications read were not all very first rate, it must be remembered that the committee appointed to winnow the chaff from the wheat appeared to have taken too good-natured a view, and to have left their "gift of criticism" behind them.

There can be no doubt, and we are pleased to find our professional brethren of America were the first to notice the matter, that much really valuable material was crowded out by the intrusion of silly papers and vapid discussions.

As far as English dentistry is concerned, it is not going too far to say that we were not represented. No really important paper was offered by an English dentist, dealing in an exhaustive way with professional matters, and the leaders of the English profession held back in a most regrettable manner from the Congress.

A feature of our section which interested all, and imparted a practical relief after some of the rather slow "talkie talkie," was the establishment of clinics. No class of professional entertainment can compare with that afforded by seeing another actually engaged upon some new method or unfamiliar development of technique. One often reads, with wide-opened eyes and mouth agape, of prodigious developments of American dental art, and all of us were more anxious to witness with our own eyes the actual practice than to hear the theory of "how to do it." After all, an ounce of practice, when it is dental, is worth a hundred-weight of precept. Clinics are what we all have need of, and it would spare us a good deal of valuable time if every one who brought a new procedure before a society should be bound to demonstrate upon a patient the practicability of his theory.

One other lesson taught by the Congress needs comment. Although in London we possessed an Oral Section at the Congress of 1881, the dentists were left out in the cold at Copenhagen in 1884, so that the undoubted success of a Dental Section in 1887 gives earnest of its continuation in subsequent meetings of the International Medical Congress.

After much dispute and many rather silly papers anent the subject, "Is Dentistry a Branch of Medicine?" the American mind has settled down into an affirmative state, and there we trust it will rest.

In England, with perhaps a few exceptions, dentists regard themselves as part of the brotherhood of the healing art, and are so received by the doctors. As time goes on, most dentists will bear registrable medical diplomas over and above the L. D. S., and then the matter will be settled. For the present, a few storms stirred up in teacups are amusing, and break the monotony of dental life.

The Congress of 1887 is over; but friendships made, debts of hospitality incurred, are like chains of roses, unbreakable by their very nature, and will, we trust, prove sempiternal. When America comes to England, let us not be wanting in efforts to show her how heartily we appreciate all her kindnesses, her hospitality and courtesy.—*Brit. Jour. Dental Science.*

### ADMINISTERING ANÆSTHETICS.

There are many who will bear witness that we have constantly urged that the chief danger in the administration of chloroform and ether is the liability to force upon the patient an atmosphere too highly charged with the vapor A more unscientific and dangerous way than that which is usually followed could scarcely be devised. What judicious physician administers powerful drugs by guess, and in almost total ignorance of the strength of the solution? Imagine any one giving opium in this way. And yet, chloroform, a drug of greater power, is constantly administered in a hap-hazard fashion.

We are impelled to these remarks by an examination of the Hayes' apparatus, advertised in this number. Of their "Hypnotic" we have no knowledge, but we do believe that this is the first apparatus yet presented to the public which will enable the administrator to have under intelligent control the vaporization of the agent—to know just the percentage in the atmosphere breathed, and to be able to increase or diminish it at will.

### NOTICE.

I am in receipt of a circular announcing the opening of "The Robinson Tooth Crown College," with my name appended as a reference "by permission." I have only to say that I know nothing about any such "college," have never been consulted concerning it, and have never authorized the use of my name as a reference. G. L. CURTISS, Syracuse, N. Y.

No one at No. 208 Franklin Street, Buffalo, has authorized the use of his name as a reference, or has any knowledge of such an institution. The circular would seem to be a deliberate piece of ——— assurance. W. C. BARRETT.

### MISNOMERS IN CHEMISTRY.

Oil of vitriol is no oil, neither are oils of turpentine and kerosene Copperas is an iron compound and contains no copper. Salts of lemon is the extremely poisonous oxalic acid. Carbolic acid is not an acid, but an alcohol. Cobalt contains none of that metal, but arsenic. Soda water has no trace of soda, nor has sulphuric acid of sulphur. Sugar of lead has no sugar, cream of tartar has nothing of cream, nor milk of lime any milk. Oxygen means the acid maker; but hydrogen is the essential element of acids, and many contain no oxygen. German silver has no silver, and black lead no lead. Mosaic gold is only a sulphide of tin.

### ALLOY FOR GOLD SOLDERS.

Take equal parts by weight of copper, silver and zinc; melt the copper and silver in a crucible, and add the zinc in small pieces; when the blue flame of gas is thrown off, pour into an ingot and call it *alloy for gold solder.* Take of this alloy one part, and of the gold plate you are using three parts, and you will have a solder that will melt easily, flow smoothly, and will not change color in the mouth; if you want it to melt harder, put in less alloy.—*Dr. J. A. Robinson.*

### WISCONSIN STATE DENTAL SOCIETY.

The following resolutions were passed at the last annual meeting of the Wisconsin State Dental Society, and are sent to you for publication :

*Whereas*, Members of this society are threatened with suits for damages and injunctions, if certain letters patent for alleged improvments in dentistry are not recognized, the validity of which has been gravely questioned, and the right to use is wholly refused, or terms and conditions imposed, which would be a heavy tax upon the profession and the community for many years; and,

*Whereas*, It would be unjust for one or two members to bear the labor and heavy expense attendant upon determining how far the pretensions of such patentees ought to be respected; therefore,

*Resolved*, That each member of this society be requested to contributed five dollars towards a protective litigation fund, to be expended as a special committee may deem advisable in carrying out the spirit of these resolutions; and,

*Resolved*, That said fund shall be termed the "Litigation Fund," and to remain in the hands of the committee until expended, or by lack of prosecution, returnable to the different subscribers of the fund *pro rata*.

---

### CONNECTICUT VALLEY DENTAL SOCIETY.

At the annual meeting, held at Springfield, Mass., October 27th and 28th, the following officers were elected:

*President*—R. R. Andrews, of Cambridge, Mass.

*Vice-Presidents*—F. W. Williams, Greenfield, Mass.; George W. Lovejoy Montreal, P Q

*Secretary*—Geo. A. Maxfield, Holyoke, Mass.

*Treasurer*—W. F. Andrews, Springfield, Mass

---

### MASSACHUSETTS DENTAL SOCIETY.

The twenty-third annual meeting of the Massachusetts Dental Society will be held in Boston, Thursday and Friday, December 8th and 9th, 1887. The Executive Committee is doing all in its power to make this meeting a profitable one, and asks your hearty co-operation. Mark the time off *now*, and be at the meetings from start to finish. Full programmes will be sent later.

Per order Ex. Committee,

GEO. F. EAMES, Secretary.

---

*Editor Independent Practitioner:*

How can any man who comprehends English make such a misapplication of the words "former" and "latter" as that contained in the criticism upon Dr. Black's paper on "The Formation of Pus," under the caption, "Dental Squibbs," in the PRACTITIONER for November ?

The sentences criticised are as follows :—

"In this way the formation of the matrix filled with fresh ameboid cells, which tend to develop into granulations, is constantly proceeding. And the liquefaction of this exudate, carrying with it the ameboid cells in the form of pus, is also constantly proceeding. If the *former* exceed the *latter*, the healing of the wound is being accomplished; but if the *latter* exceed the *former*, destruction of tissue and widening of the breach of continuity is the result".

Here are two things, antagonistic to each other, said to be proceeding at the same time, and the words former and latter are used to designate them, respectively. The exudate (or matrix) and the ameboid cells are associated together in each of the two processes referred to; in the first as being built up into the tissue of repair, and in the second as being destroyed together in the formation of pus. It is not possible, grammatically, to apply the word *former* to "the liquefaction of this exudate" and the word *latter* to "the ameboid cells"

Yours truly,

EDMUND NOYES.

---

DR. GEO. D. HAYS, of the New York Post-Graduate School, writing in the *Quarterly Bulletin*, says: "We have long been accustomed to hear that many of the evils of modern life owe their origin to our choice of *white* flour. That this is not so, an examination of the wheat-berry will show. This has five coats—an epi- meso- and endocarp, an episperm, and a tegmen. The three outer ones have no value whatever as nutriment; within the episperm is a layer of gluten cells, chiefly albuminoids, and finally, in the endosperm, which constitutes the bulk of the grain, we find starch mixed with albuminoid cells. In the old process of milling, the perisperm (the part within the episperm) was, on account of its close attachment to the inner husk, largely carried away, leaving the bolted flour the poorer for its loss. Hence the vegetarian, Sylvester Graham, whose name is applied to bread made from unbolted flour, was correct in his time in saying such bread contained the most nutriment. The present gradual reduction process saves this portion of the wheat. The bran itself is composed of woody fibre, and contains absolutely no nutriment. It may have a mechanical value with those of a constipated tendency, but this is all. The wheat loaf and the white flour contain a much larger percentage of phosphates and gluten than the Graham loaf of unbolted flour.—*Lancet and Clinic.*

---

PARKE, DAVIS & CO., the well-known manufacturing chemists of Detroit, have ruthlessly exposed what appears to be a bare-faced fraud upon the medical profession. Stenocarpin, or Gleditschine, was ushered in with a great flourish of trumpets as a local anæsthetic of even greater power than cocaine, and was favorably received, until Parke, Davis & Co. published an analysis of the preparation, showing that it was almost impossible to obtain any alkaloid from the tree so highly vaunted, and that the preparation owed its virtues to an admixture of cocaine itself. For many years the chemical manufactures of E. R. Squibb, of Brooklyn, have been the standard of quality in pharmacy, but lately it would seem, from the opinion of many medical journals, that those of Parke, Davis & Co. are taking that position.

---

THE USE OF A BONE PEG IN AN OPERATION FOR PSEUD-ARTHROSIS.—At a recent meeting of the Paris *Société de Chirurgie*, a report of which appears in the *Deutsche Medezinal-Zeitung*, M. Richelot related the case of an hysterical girl, sixteen years old, with a congenital atrophy of the face, for which resection of a portion of the lower jaw was performed. The fragments were united with silver wire, but the patient was restless, and the union which took place was by fibrous tissue. Dr. Rontier subsequently drilled a hole through the fragments, excised the callus, and pegged the two parts of the bone together with a portion of the tibia of a calf, which had been steeped for twenty-four hours in a solution of 1 part of corrosive sublimate in a mixture of 900 parts of distilled water and 100 of alcohol. Bony union followed, with only slight asymmetry, and the patient could eat better than before. No disturbances of the dental nerves were observed —*N. Y. Med. Jour.*

---

THE UNION MEETING of the Sixth, Seventh and Eighth District Dental Societies of New York, held in Buffalo, October 25th and 26th ult., was one of the largest and most profitable meetings ever held in Western New York. The papers were of a high order of merit, and the discussions were intelligent and instructive. A banquet was given by the Eighth District Society to its guests, at The Genesee, on the evening of the 26th, and it was emphatically one of those affairs which bring men nearer to each other in more ways than one. A report of the meeting was expected for this number, but it did not materialize. Several of the papers read will be published by the INDEPENDENT PRACTITIONER, that of Dr. Rishel appearing in this number.

---

BRIDGE-WORK is sweeping into the profession with lightning speed, and the next four years will see many noble successes, and many lamentable failures. It behooves us, as sensible men, to stand as a unit against its unwarranted use.

First seized upon by a class of men of a mercenary spirit, it was inserted in every case where there remained a few old stumps, provided the patient was willing to pay for the experiment. The reputable practitioner is a gainer in this one direction, that they caused the idea to spread by the liberal use of printer's ink, and thus brought to many, who would otherwise never have heard of it, the knowledge that teeth could be inserted without a plate.—*Cin Med. and Dental Journal.*

---

TWENTY YEARS AGO a dentist, now dead, who bore an honored name, and whose memory is yet revered wherever he was known, said to us that when we wished any of the precious metals prepared by a man in whose thorough honesty and fidelity the most implicit confidence could be reposed, we should go to Thomas Dennis, of Buffalo. From that time to the present he has prepared our gold plate and solders, and beaten much of the foil we have used, and our experience amply confirms that of our deceased friend. Mr. Dennis still continues the business, as may be learned by consulting his advertisement in this number.

---

DR. H. C. MERRIAM says that a tooth is worth itself, the teeth with which it occludes, and all that they united can do for the organization. Not long since a lady of eighteen years of age had the toothache, and the so-called dentist whom she consulted extracted three lower molars on one side. That person was crippled for life for that one toothache. It makes one burn with indignation to hear such practice called dentistry, and such a man called a dentist.—*National Druggist.*

GOLD WILL ONLY MELT at a comparatively high temperature, as we all know, but what is not generally known, *The Jeweler's Journal* says, is that if ten per cent. of silica be added to the gold, it can be melted over the flame of a common candle.

From the same source the reader may learn that a pretty alloy, said to resemble gold exactly, can be made with sixteen parts of copper, one of zinc, and seven of platinum. The copper and platinum are covered first with borax, and then with powdered charcoal and melted, then the zinc added, and the alloy thus produced is exceedingly malleable, and can be drawn into the finest wire, while it never tarnishes.—*Scientific American.*

This item comes from a high source, but in our opinion it will bear careful tests before being put to practical use.—EDITOR.

---

FOR THE MAKING of a good amalgam pure mercury is essential It is usually adulterated with lead, and if any one wishes to know what kind of an alloy a proportion of lead will make, he has but to add some of it to the mass.

To remove the lead from mercury, place it in a shallow vessel, cover it with one part of chemically pure nitric acid to three or four parts of water, and allow it to stand two hours, stirring it frequently. Then wash out the acid and bottle for use.

A good amalgam should possess the following properties:

| | |
|---|---|
| 1. Ready amalgamation. | 4. Non-expansion. |
| 2. Moderately quick setting. | 5. Good edge strength. |
| 3. Non-shrinkage. | 6. High color standard. |

---

IN THE REPORT of the Quebec meeting of the Connecticut Valley Dental Society, in this number, will be found some references to Dr. Stevens' Disk Cutters, and in the advertising pages it will be seen that they are now manufactured and placed on sale by Dr. Stevens. For a number of years we have had in use a set of these cutters, and every disk used in our office—and they number many thousands—has been prepared by the office girl with little trouble and at no appreciable expense. There is not an instrument, in either operating case or laboratory, from which has been received better returns, in both economy and satisfaction, than from the Stevens Disk Cutter.

---

DR. VON WEDEKIND, senior physician at the Chambers Street Hospital, New York, recommends pressure upon the supra-orbital notch with the point of the thumb steadily applied and constantly increased for thirty seconds, the patient being in a recumbent position, as a means to distinguish between malingerers, drunken men, hysterical persons, etc., and those who are suffering from serious injuries. It is impossible for those who are not in a state of coma to withstand this test, and those who are in a state of alcoholic insensibility are invariably brought to consciousness. Try the pressure over your own eye.

---

HEBRA'S OINTMENT, of equal parts of linseed oil and diachylon ointment, prepared with the aid of heat, is recommended as a certain cure for hyperideosis, or excessive sweating of the feet.

Mrs. Cunningham, who thirty years ago was tried for the murder of Dr. Harvey Burdell, a well-known dentist of New York City, and who was acquitted by the jury, though believed to be guilty by the majority of those acquainted with the circumstances, died recently in New York, while on a visit from California, where she had long been a resident, and where she had married a man of wealth and position, named Williams. The Burdell murder case convulsed the whole country at the time of the trial, and has taken its place in history as one of the great cases of the century. The woman for many years had led a quiet and peaceable life.

---

Archives of Pediatrics, published by J. B. Lippincott Company, of Philadelphia, with the number for January, 1888, will begin the publication of a series of articles on the Therapeutics of Infancy and Childhood, by A. Jacobi, M. D., Clinical Professor of Diseases of Children in the College of Physicians and Surgeons; President of the New York Academy of Medicine, etc. The subject will commend itself to the attention of every physician, and one more competent than Professor Jacobi to write upon the theme could not be found.

---

Within the last fifteen years the curious discovery has been made, that the earth's surface is being continually shaken by tremors so minute as to remain unsuspected without the intervention of the most delicate instruments. In every country where the experiment has been tried these tremors have been detected, and not merely at certain periods, but so incessantly that there is never a second of perfect rest. The earth may fairly be said to tremble like a jelly.

---

Creosote is a distillation from wood tar, carbolic acid from tar of mineral coal; creosote is an oil, carbolic acid an alcohol; creosote is a non-crystallizable fluid, carbolic acid in its pure state is always crystallized, except when quite warm; creosote is not soluble in water, carbolic acid is; creosote is not caustic, carbolic acid is a powerful caustic; creosote is not a germicide, carbolic acid is.
—*Mat. Med. and Pharm.*

---

The Christmas Number of *Scribner's Magazine* contains double the usual number of illustrations, every one of which has been made from a drawing by some well-known and expert artist. A few of those represented are Will H. Low, William Hole, A. R. S. A., R. Swain Gifford, Howard Pyle, E. H. Blashfield, J. W. Alexander, George Foster Barnes, F. Hopkinson Smith and F. S. Church. The price will remain the same as usual—25 cents.

---

"The Toothache," says Roger Williams, in his observations upon the language and customs of the New England tribes of Indians, "is the only paine which will force their stout hearts to cry." He afterwards remarks that even the Indian women never cry as he has heard "some of their men in this paine."—*Notes to Whittier's Mogg Megone.*

---

A student in the Albany Medical College when asked how he would treat a corpulent man, replied that he had found in an extensive experience that such men usually take beer.

ARSENIC WILL NOT cause pain when combined with carbolic acid if there be no pressure upon the exposed pulp. If applied in heroic doses it is a caustic, but in infinitesimal amounts and combined with carbolic acid it is a sedative. It assuages pain immediately, and it subdues inflammation. It is the pressure on the inflamed pulp that causes the pain brought on by a bungling application. I never put anything like gutta-percha over the application, but lay cotton gently over the opening to the pulp, and sometimes tie a thread about it to hold it in place. I use pure carbolic acid to paralyze the pulp while the arsenic devitalizes it. DR. J. A. ROBINSON.

---

SINCE THE READING, last summer, of the paper on Copper Amalgam, published in this number, Dr Weagant has found himself so overwhelmed with requests and orders for it that he has been obliged, in self-defense, to place it upon the market, and his advertisement will be found in the advertising pages. We have tested it, and are delighted with its plasticity and beautiful working qualities.

---

DURING THE REIGN of Henry VIII, there were twelve surgeons in London. In 1795, the number of physicians in London was but ninety-four. The apothecaries, not including surgeons, amounted to 4,000.

In 1512, physicians and surgeons had to be approved by the Bishop of London or the Dean of St. Paul's. Females were everywhere to be met with practicing the healing art.—*Medical Classics.*

---

THE MOST ancient recipes on record are those mentioned in the Pentateuch for preparation of an odoriferous ointment and confection. Their date is 1491 B C.

The work of Scribonius Largus, who lived about the middle of the first century of the Christian era, is a collection of recipes taken from various authors. It is the oldest pharmacopœia extant, but its style is very inelegant —*Medical Classics.*

---

THE CHEAPEST and simplest gymnasium in the world—one that will exercise every bone and muscle in the body—is a flat piece of steel notched on one side, fitted firmly into a wooden frame. After being greased with a bacon rind it is rubbed into a stick of wood laid lengthwise of a saw-buck.—*Medical Times.*

---

"PROF JOHN THOMAS JOHNSON, M. D , PH.D., F. C S., REGISTRAR KINGSVILLE COLLEGE OF DENTISTRY," is the way in which it appears printed upon the envelopes enclosed for a return letter. The man who will address himself in that way cannot be entirely destitute of assurance.

---

IT IS A GENERAL IMPRESSION that the editor of a journal must have some acquaintance with grammar, and be able to write intelligible and intelligent English. This has been proved a fallacy by *The Medical Brief*, a new monthly recently established at Lafayette, Ind.

---

THE AREA of the United States is 2,970,000 square miles, and Burdette says it is no wonder a fellow has such on all-howling time finding his collar-button when it rolls out of sight.

## Figures and Tables

**Figure f1:**
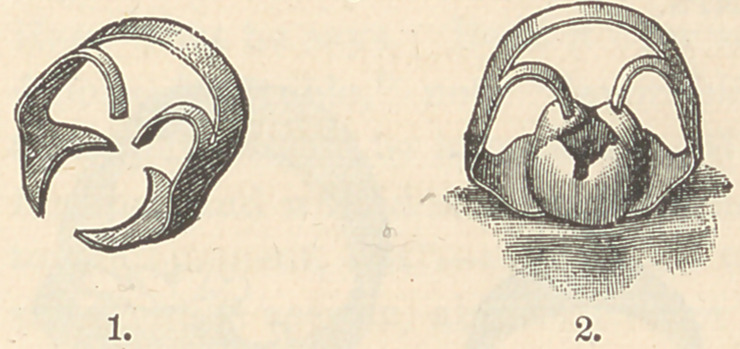


**Figure f2:**